# Supplementary material for: Personalized customization: Service resource configuration optimization driven by customer requirements accurately
Source: PLoS One. 2025 Apr 14;20(4):e0320312. doi: 10.1371/journal.pone.0320312 (PMC11996078; doi:10.1371/journal.pone.0320312)
Supplement: S1 File — (DOCX) [file pone.0320312.s001.docx]

**Table 6** The hierarchical division of service modules and candidate itemsets

| service module | candidate itemset | candidate itemset interpretation |
| --- | --- | --- |
| Air Conditioner selection  ($SM_{1}$) | $SC_{1}^{1}=\left\{SC_{11}^{1},SC_{12}^{1},...,SC_{17}^{1}\right\}$ | HP={ WM-1hp,WM-1.5hp,V-1.5hp,V-2hp,V-3hp,C-3hp,C-4hp} |
|  | $SC_{1}^{2}=\left\{SC_{11}^{2}\right\}$ | Adjust humidity range ={65%} |
|  | $SC_{1}^{5}=\left\{SC_{11}^{5},SC_{12}^{5},SC_{13}^{5}\right\}$ | energy efficiency index ={ Level 1-4.78, Level 2-4.19, Level 3-3.99 } |
| Heating equipment selection（$SM_{2}$） | $SC_{2}^{1}=\left\{SC_{21}^{1},SC_{22}^{1},...,SC_{\text{2（10）}}^{1}\right\}$ | Watt={E-2000W,E-2100W,E-2200W,E-3000W,R-1800W,R-1900W,  R-2000W,R-2100W,R-2200W,R-2440W} |
| Window selection  （$SM_{3}$） | $SC_{3}^{123}=\left\{SC_{31}^{123},SC_{32}^{123},...,SC_{35}^{123}\right\}$ | Window area ={4.5$\text{m}^{2}$,5$\text{m}^{2}$,5.5$\text{m}^{2}$,6$\text{m}^{2}$,7$\text{m}^{2}$} |
|  | $SC_{3}^{6}=\left\{SC_{31}^{6},SC_{32}^{6},...,SC_{36}^{6}\right\}$ | Sound insulation decibel={25dB,26dB,27dB,28dB,29dB,30dB} |
| Flooring selection  （$SM_{4}$） | $SC_{4}^{2}=\left\{SC_{41}^{2},SC_{42}^{2},SC_{43}^{2}\right\}$ | Floor material ={ marble, Strengthening compound, Solid wood composite } |
| Interior paint selection  （$SM_{5}$） | $SC_{5}^{4}=\left\{SC_{51}^{4},SC_{52}^{4},SC_{53}^{4}\right\}$ | Environmental protection level={France-A+,USA-GREENGUARD，ACEM-10} |
| Lamps and lighting design  （$SM_{6}$） | $SC_{6}^{3}=\left\{SC_{61}^{3},SC_{62}^{3},...,SC_{66}^{3}\right\}$ | Illuminance ={200lx,280lx,330lx,350lx,450lx,500lx} |
|  | $SC_{6}^{5}=\left\{SC_{61}^{5},SC_{62}^{5},...,SC_{65}^{5}\right\}$ | Luminous efficacy ={70lm/W,90 lm/W,100 lm/W,110 lm/W,130 lm/W } |
| Ceiling material selection  （$SM_{7}$） | $SC_{7}^{4}=\left\{SC_{71}^{4},SC_{72}^{4},SC_{73}^{4}\right\}$ | Ceiling materials ={PVC，Aluminum Alloy，Gypsum Board } |
|  | $SC_{7}^{6}=\left\{SC_{71}^{6},SC_{72}^{6},...,SC_{74}^{6}\right\}$ | Sound insulation decibel ={46dB,47dB,48dB,49dB} |
| Cabinet customization  （$SM_{8}$） | $SC_{8}^{2}=\left\{SC_{81}^{2},SC_{82}^{2},SC_{83}^{2}\right\}$ | Plate={ Particle board, Plywood, Ecological board } |
|  | $SC_{8}^{4}=\left\{SC_{81}^{4},SC_{82}^{4},SC_{83}^{4}\right\}$ | Environmental protection level ={ENF,E0,E1} |
| Color temperature adjustment  （$SM_{9}$） | $SC_{9}^{3}=\left\{SC_{91}^{3},SC_{92}^{3}\right\}$ | Dimming method ={ Three-color dimming mode, Stepless dimming mode } |

**Table 8** The cost of each candidate item configuration scheme in the existing service module

| Service modules | Feasible candidate item configuration scheme | Corresponding cost |
| --- | --- | --- |
| $SM_{1}$ | $OC_{11}^{*}=\left\{SC_{11}^{1},SC_{11}^{2},SC_{11}^{5}\right\}$,$OC_{12}^{*}=\left\{SC_{12}^{1},SC_{11}^{2},SC_{11}^{5}\right\}$,$OC_{13}^{*}=\left\{SC_{13}^{1},SC_{11}^{2},SC_{11}^{5}\right\}$,$OC_{14}^{*}=\left\{SC_{14}^{1},SC_{11}^{2},SC_{11}^{5}\right\}$,$OC_{15}^{*}=\left\{SC_{15}^{1},SC_{11}^{2},SC_{11}^{5}\right\}$,$OC_{16}^{*}=\left\{SC_{16}^{1},SC_{11}^{2},SC_{11}^{5}\right\}$,$OC_{17}^{*}=\left\{SC_{17}^{1},SC_{11}^{2},SC_{11}^{5}\right\}$,$OC_{18}^{*}=\left\{SC_{15}^{1},SC_{11}^{2},SC_{12}^{5}\right\}$,$OC_{19}^{*}=\left\{SC_{16}^{1},SC_{11}^{2},SC_{12}^{5}\right\}$,$OC_{1(10)}^{*}=\left\{SC_{11}^{1},SC_{11}^{2},SC_{13}^{5}\right\}$,$OC_{1(11)}^{*}=\left\{SC_{12}^{1},SC_{11}^{2},SC_{13}^{5}\right\}$,$OC_{1(12)}^{*}=\left\{SC_{13}^{1},SC_{11}^{2},SC_{13}^{5}\right\}$,$OC_{1(13)}^{*}=\left\{SC_{14}^{1},SC_{11}^{2},SC_{13}^{5}\right\}$,$OC_{1(14)}^{*}=\left\{SC_{15}^{1},SC_{11}^{2},SC_{13}^{5}\right\}$,$OC_{1(15)}^{*}=\left\{SC_{16}^{1},SC_{11}^{2},SC_{13}^{5}\right\}$. | $c_{11}=1799$,$c_{12}=2049$,$c_{13}=2649$,$c_{14}=4169$,$c_{15}=5108$,$c_{16}=5979$,$c_{17}=8499$,$c_{18}=4899$,$c_{19}=5799$,$c_{1(10)}=1599$,$c_{1(11)}=1844$,$c_{1(12)}=1799$,$c_{1(13)}=3669$,$c_{1(14)}=4669$,$c_{1(15)}=5589$. |
| $SM_{2}$ | $OC_{21}^{*}=\left\{SC_{21}^{1}\right\}$,$OC_{22}^{*}=\left\{SC_{22}^{1}\right\}$,$OC_{23}^{*}=\left\{SC_{23}^{1}\right\}$,$OC_{24}^{*}=\left\{SC_{24}^{1}\right\}$,$OC_{25}^{*}=\left\{SC_{25}^{1}\right\}$,$OC_{26}^{*}=\left\{SC_{26}^{1}\right\}$,$OC_{27}^{*}=\left\{SC_{27}^{1}\right\}$,$OC_{28}^{*}=\left\{SC_{28}^{1}\right\}$,$OC_{29}^{*}=\left\{SC_{29}^{1}\right\}$,$OC_{2(10)}^{*}=\left\{SC_{2\left(10\right)}^{1}\right\}$. | $c_{21}=219$,$c_{22}=399$,$c_{23}=499$,$c_{24}=549$,$c_{25}=879$,$c_{26}=929$,$c_{27}=979$,$c_{28}=1029$,$c_{29}=1075$,$c_{2(10)}=1192$. |
| $SM_{3}$ | $OC_{3s}^{*}=\left\{SC_{3j^{123}}^{123},SC_{3j^{6}}^{6}\right\}$ | $c_{3s}=area\times unit\text{ }price$ |
| $SM_{4}$ | $OC_{41}^{*}=\left\{SC_{41}^{2}\right\}$,$OC_{42}^{*}=\left\{SC_{42}^{2}\right\}$,$OC_{43}^{*}=\left\{SC_{43}^{2}\right\}$. | With a loss rate of 5%, a total of 31.5$m^{2}$ are required, and the cost is determined accordingly.  $c_{41}=4410$,$c_{42}=3433.5$,$c_{43}=3150$. |
| $SM_{5}$ | $OC_{51}^{*}=\left\{SC_{51}^{4}\right\}$,$OC_{51}^{*}=\left\{SC_{52}^{4}\right\}$,$OC_{53}^{*}=\left\{SC_{53}^{4}\right\}$. | The capacity of each barrel is 5 liters, and the required quantity is 5 barrels. Therefore, the cost is determined accordingly.  $c_{51}=1395$,$c_{52}=930$,$c_{53}=690$. |
| $SM_{6}$、$SM_{9}$ | $OC_{61}^{*}=\left\{SC_{66}^{3},SC_{65}^{5},SC_{92}^{3}\right\}$,$OC_{62}^{*}=\left\{SC_{65}^{3},SC_{64}^{5},SC_{92}^{3}\right\}$,$OC_{63}^{*}=\left\{SC_{64}^{3},SC_{63}^{5},SC_{91}^{3}\right\}$, $OC_{64}^{*}=\left\{SC_{63}^{3},SC_{62}^{5},SC_{91}^{3}\right\}$,$OC_{65}^{*}=\left\{SC_{62}^{3},SC_{62}^{5},SC_{91}^{3}\right\}$, $OC_{66}^{*}=\left\{SC_{61}^{3},SC_{61}^{5},SC_{91}^{3}\right\}$. | $c_{61}=719$,$c_{62}=699$,$c_{63}=649$,$c_{64}=433$,$c_{65}=527$,$c_{66}=369$. |
| $SM_{7}$ | $OC_{71}^{*}=\left\{SC_{71}^{4},SC_{71}^{6}\right\}$,$OC_{72}^{*}=\left\{SC_{72}^{4},SC_{71}^{6}\right\}$,$OC_{73}^{*}=\left\{SC_{72}^{4},SC_{74}^{6}\right\}$,$OC_{74}^{*}=\left\{SC_{73}^{4},SC_{72}^{6}\right\}$,$OC_{75}^{*}=\left\{SC_{73}^{4},SC_{74}^{6}\right\}$. | With a loss rate of 10%, a total of 30.3$m^{2}$ are required, and the cost is determined accordingly.  $c_{71}=1212$,$c_{72}=3333$,$c_{73}=5454$,$c_{74}=3333$,$c_{75}=4545$. |
| $SM_{8}$ | $OC_{81}^{*}=\left\{SC_{81}^{2},SC_{81}^{4}\right\}$,$OC_{82}^{*}=\left\{SC_{81}^{2},SC_{82}^{4}\right\}$,$OC_{83}^{*}=\left\{SC_{82}^{2},SC_{82}^{4}\right\}$,$OC_{84}^{*}=\left\{SC_{82}^{2},SC_{83}^{4}\right\}$,$OC_{85}^{*}=\left\{SC_{83}^{2},SC_{81}^{4}\right\}$,$OC_{86}^{*}=\left\{SC_{83}^{2},SC_{82}^{4}\right\}$,$OC_{87}^{*}=\left\{SC_{83}^{2},SC_{83}^{4}\right\}$. | Approximately 10 sheets of board are expected to be utilized, and the cost will be determined accordingly.  $c_{81}=2290$，$c_{82}=1750$，$c_{83}=2200$，$c_{84}=2060$，$c_{85}=2980$，$c_{86}=2460$，$c_{87}=2190$ |

*In $SM_{3}$, $SC_{3j^{123}}^{123}$ is the window area, its unit is $m^{2}$;$SC_{3j^{6}}^{6}$ is the window sound insulation effect, its unit price is RMB$\text{/}m^{2}$,so when calculating the cost of the feasible candidate configuration item scheme $OC_{3s}^{*}$ in $SM_{3}$,the formula is: $c_{3s}=area\times unit\text{ }price$.

The customer requirements importance judgment matrix is obtained as follows:

$$Z\text{=}\left[\begin{matrix} 1 & 2 & 3 & \frac{1}{3} & 3 & 3 \\ \frac{1}{2} & 1 & 2 & \frac{1}{3} & \frac{1}{2} & 1 \\ \frac{1}{3} & \frac{1}{2} & 1 & \frac{1}{3} & 2 & 1 \\ 3 & 3 & 3 & 1 & 3 & 3 \\ \frac{1}{3} & 2 & \frac{1}{2} & \frac{1}{3} & 1 & 2 \\ \frac{1}{3} & 1 & 1 & \frac{1}{3} & \frac{1}{2} & 1 \end{matrix}\right]$$

**Table 9** The specific results of $w_{l}^{LCI}$

| CR | AR | OR | MR | IR | RR | AI | $w_{l}^{LCI}$ |
| --- | --- | --- | --- | --- | --- | --- | --- |
| $CR_{1}$ | 27 | 47 | 74 | 0 | 0 | 538 | 0.2036 |
| $CR_{2}$ | 62 | 31 | 23 | 32 | 0 | 270 | 0.1022 |
| $CR_{3}$ | 40 | 63 | 45 | 0 | 0 | 454 | 0.1718 |
| $CR_{4}$ | 24 | 39 | 85 | 0 | 0 | 566 | 0.2142 |
| $CR_{5}$ | 17 | 87 | 28 | 16 | 0 | 418 | 0.1583 |
| $CR_{6}$ | 22 | 73 | 31 | 22 | 0 | 396 | 0.1499 |

**Table 10** The specific market competitiveness evaluation results

| CR | $p_{l}$ | $p_{l}^{'}$ | $w_{l}^{P}$ |
| --- | --- | --- | --- |
| $CR_{1}$ | 5 | 5 | 1.00 |
| $CR_{2}$ | 4 | 5 | 1.25 |
| $CR_{3}$ | 4 | 5 | 1.25 |
| $CR_{4}$ | 5 | 5 | 1.00 |
| $CR_{5}$ | 4 | 5 | 1.25 |
| $CR_{6}$ | 3 | 4 | 1.33 |

**Table 11** The calculation results of $w_{l}$

| CR | $w_{l}^{Z}$ | $w_{l}^{LCI}$ | $w_{l}^{P}$ | $w_{l}'$ | $w_{l}$ |
| --- | --- | --- | --- | --- | --- |
| $CR_{1}$ | 0.2319 | 0.2036 | 1.00 | 4.7214 | 0.24 |
| $CR_{2}$ | 0.1063 | 0.1022 | 1.25 | 1.3580 | 0.07 |
| $CR_{3}$ | 0.0993 | 0.1718 | 1.25 | 2.1325 | 0.11 |
| $CR_{4}$ | 0.3579 | 0.2142 | 1.00 | 7.6662 | 0.38 |
| $CR_{5}$ | 0.1115 | 0.1583 | 1.25 | 2.2063 | 0.11 |
| $CR_{6}$ | 0.0885 | 0.1499 | 1.33 | 1.7644 | 0.09 |

The correlation matrix based on QFD provided by the service design team is as follows:

$R^{1}=\left[\begin{matrix} 5 & 0 & 0 & 0 & 0 & 3 & 0 & 0 \\ 0 & 3 & 5 & 0 & 0 & 5 & 0 & 0 \\ 0 & 0 & 0 & 0 & 5 & 3 & 0 & 0 \\ 0 & 0 & 0 & 0 & 0 & 0 & 5 & 0 \\ 0 & 0 & 0 & 0 & 0 & 0 & 0 & 5 \\ 0 & 0 & 0 & 5 & 0 & 0 & 0 & 0 \end{matrix}\right]$ $R^{2}=\left[\begin{matrix} 5 & 5 & 0 & 0 & 0 & 0 & 0 & 0 & 0 \\ 3 & 0 & 0 & 0 & 0 & 0 & 0 & 0 & 0 \\ 0 & 0 & 3 & 3 & 0 & 0 & 0 & 5 & 0 \\ 0 & 0 & 3 & 0 & 0 & 0 & 3 & 0 & 0 \\ 0 & 0 & 0 & 0 & 0 & 5 & 0 & 0 & 5 \\ 0 & 0 & 3 & 0 & 0 & 0 & 0 & 0 & 0 \\ 0 & 0 & 0 & 0 & 5 & 0 & 3 & 3 & 0 \\ 5 & 0 & 0 & 0 & 0 & 3 & 0 & 0 & 0 \end{matrix}\right]$

**Table 12** The utility measure function of the candidate itemsets

| $SM$ | $SC_{i}^{l}$ | utility measure functions |
| --- | --- | --- |
| $SM_{1}$ | $SC_{1}^{1}$ | $f^{1}(b_{1j}^{1})=\left\{\begin{matrix} 0, & b_{1j}^{1}<2 \\ 1, & b_{1j}^{1}\geq2 \end{matrix}\right.$ |
|  | $SC_{1}^{2}$ | $f^{4}(b_{1j}^{2})=\left\{\begin{matrix} 0, & b_{1j}^{2}<30\% \\ \sin\frac{\pi}{2(70\%-30\%)}(b_{1j}^{2}-30\%) & 30\%\leq b_{1j}^{2}<70\% \\ 1 & b_{1j}^{2}\geq\text{70\%} \end{matrix}\right.$ |
|  | $SC_{1}^{5}$ | $f^{4}(b_{1j}^{5})=\left\{\begin{matrix} 0, & b_{1j}^{5}<3.50 \\ \sin\frac{\pi}{2(4.50-3.50)}(b_{1j}^{5}-3.50) & 3.50\leq b_{1j}^{5}<4.50 \\ 1 & b_{1j}^{5}\geq4.50 \end{matrix}\right.$ |
| $SM_{2}$ | $SC_{2}^{1}$ | $f^{4}(b_{2j}^{1})=\left\{\begin{matrix} 0, & b_{2j}^{1}<1800 \\ \sin\frac{\pi}{2(2200-1800)}(b_{2j}^{1}-1800) & 1800\leq b_{2j}^{1}<2200 \\ 1 & b_{2j}^{1}\geq2200 \end{matrix}\right.$ |
| $SM_{3}$ | $SC_{3}^{1}$ $SC_{3}^{2}$ $SC_{3}^{3}$ | $f^{5}(b_{3j}^{1})=f^{5}(b_{3j}^{2})=f^{5}(b_{3j}^{3})=\left\{\begin{matrix} 0\text{，} & b_{3j}^{1}<4.3 \\ \sin\frac{\pi}{2(5-4.3)}(b_{3j}^{1}-4.3), & 4.3\leq b_{3j}^{1}<5 \\ 1, & 5\leq b_{3j}^{1}<7.5 \\ -\sin\frac{\pi}{2(9.8-7.5)}(b_{3j}^{1}-9.8), & 7.5\leq b_{3j}^{1}<9.8 \\ 0, & b_{3j}^{1}\geq9.8 \end{matrix}\right.$ |
|  | $SC_{3}^{6}$ | $f^{4}(b_{3j}^{6})=\left\{\begin{matrix} 0, & b_{3j}^{6}<25 \\ \sin\frac{\pi}{2(30-25)}(b_{3j}^{6}-25) & 25\leq b_{3j}^{6}<30 \\ 1 & b_{3j}^{6}\geq30 \end{matrix}\right.$ |
| $SM_{4}$ | $SC_{4}^{2}$ | $f^{3}(b_{4j}^{2})=\left\{\begin{matrix} \text{0.6，} & \text{ }b_{4j}^{2}\text{=}Solid\text{ }wood\text{ }composite \\ \text{0.8，} & b_{4j}^{2}\text{=}Strengthening\text{ }compound \\ \text{1，} & b_{4j}^{2}\text{=}marble \end{matrix}\right.$ |
| $SM_{5}$ | $SC_{5}^{4}$ | $f^{3}(b_{5j}^{4})=\left\{\begin{matrix} \text{0，} & \text{ }b_{5j}^{4}\text{=Third-party appraisal agency} \\ \text{0.6，} & b_{5j}^{4}\text{=}ACEM:(10) \\ \text{0.8，} & b_{5j}^{4}\text{=}USA:GREENGUARD \\ \text{1，} & b_{5j}^{4}\text{=}France:A+ \end{matrix}\right.$ |
| $SM_{6}$ | $SC_{6}^{3}$ | $f^{5}(b_{6j}^{3})=\left\{\begin{matrix} 0\text{，} & b_{6j}^{3}<100 \\ \sin\frac{\pi}{2(300-100)}(b_{6j}^{3}-100), & 100\leq b_{6j}^{3}<300 \\ 1, & 300\leq b_{6j}^{3}<500 \\ -\sin\frac{\pi}{2(1000-500)}(b_{6j}^{3}-1000), & 500\leq b_{6j}^{3}<1000 \\ 0, & b_{6j}^{3}\geq1000 \end{matrix}\right.$ |
|  | $SC_{6}^{5}$ | $f^{4}(b_{6j}^{5})=\left\{\begin{matrix} 0, & b_{6j}^{5}<50 \\ \sin\frac{\pi}{2(130-50)}(b_{6j}^{5}-50) & 50\leq b_{6j}^{5}<130 \\ 1 & b_{6j}^{5}\geq130 \end{matrix}\right.$ |
| $SM_{7}$ | $SC_{7}^{4}$ | $f^{3}(b_{7j}^{4})=\left\{\begin{matrix} \text{0.6，} & \text{ }b_{7j}^{4}\text{=PVC} \\ \text{0.8，} & b_{7j}^{4}\text{=}Aluminum\text{ }Alloy \\ \text{1，} & b_{7j}^{4}\text{=}Gypsum\text{ }Board \end{matrix}\right.$ |
|  | $SC_{7}^{6}$ | $f^{4}(b_{7j}^{6})=\left\{\begin{matrix} 0, & b_{7j}^{6}<45 \\ \sin\frac{\pi}{2(50-45)}(b_{7j}^{6}-45) & 45\leq b_{7j}^{6}<50 \\ 1 & b_{7j}^{6}\geq50 \end{matrix}\right.$ |
| $SM_{8}$ | $SC_{8}^{2}$ | $f^{3}(b_{8j}^{2})=\left\{\begin{matrix} \text{0.6，} & \text{ }b_{8j}^{2}\text{=}Particle\text{ }board \\ \text{0.8，} & b_{8j}^{2}\text{=}Plywood \\ \text{1，} & b_{8j}^{2}\text{=}Ecological\text{ }board \end{matrix}\right.$ |
|  | $SC_{8}^{4}$ | $f^{3}(b_{8j}^{4})=\left\{\begin{matrix} \text{0.6，} & \text{ }b_{8j}^{4}\text{=E1} \\ \text{0.8，} & b_{8j}^{4}\text{=E0} \\ \text{1，} & b_{8j}^{4}\text{=ENF} \end{matrix}\right.$ |
| $SM_{9}$ | $SC_{9}^{3}$ | $f^{2}(b_{9j}^{3})=\left\{\begin{matrix} 0,\text{ }b_{9j}^{3}\not\subset\left\{warm\text{ }light\text{，}Two-color\text{，}Three-color\text{ },\text{ }Stepless\text{ }\right\} \\ 1,\text{ }b_{9j}^{3}\subseteq\left\{warm\text{ }light\text{，}Two-color\text{，}Three-color\text{ },\text{ }Stepless\right\} \end{matrix}\right.$ |

**Table 14** The optimal parameter combination and model solution results

| DL | PS | NG | MR | MCS^*^ | $q$ | $c_{sum}$ |
| --- | --- | --- | --- | --- | --- | --- |
| 43 | 300 | 100 | 0.1 | $\begin{matrix} SC_{14}^{1},SC_{11}^{2},SC_{11}^{5},SC_{23}^{1},SC_{32}^{123},SC_{32}^{6},SC_{43}^{2},SC_{51}^{4}, \\ SC_{63}^{3},SC_{62}^{5},SC_{73}^{4},SC_{72}^{6},SC_{83}^{2},SC_{81}^{4},SC_{91}^{3} \end{matrix}$ | 0.9328 | 24759 |

The results of the cost sensitivity analysis are as follow:

| Cost(RMB) | Fitness |
| --- | --- |
| 20000 | 0.757082851 |
| 21000 | 0.750180732 |
| 22000 | 0.863827122 |
| 23000 | 0.791295625 |
| 24000 | 0.795180157 |
| 25000 | 0.796875146 |
| 26000 | 0.871508827 |
| 27000 | 0.835267129 |
| 28000 | 0.871112524 |
| 29000 | 0.90321111 |
| 30000 | 0.948822648 |
| 31000 | 0.939978537 |
| 32000 | 0.974258811 |
| 33000 | 0.992327856 |
| 34000 | 0.935598471 |
| 35000 | 0.968107472 |
| 36000 | 0.958712698 |
| 37000 | 0.946106808 |
| 38000 | 0.916747812 |
| 39000 | 0.947486342 |
| 40000 | 0.961407014 |
