# Supplementary material for: Personalized customization: Service resource configuration optimization driven by customer requirements accurately
Source: PLoS One. 2025 Apr 14;20(4):e0320312. doi: 10.1371/journal.pone.0320312 (PMC11996078; doi:10.1371/journal.pone.0320312)
Supplement: Attachment — Submitted filename: Response to Reviewers 1-3 on PONE-D-24-37417.doc [file pone.0320312.s004.doc]

**Response to the Comments of Reviewer #1 on** **PONE-D-24-37417**

We thank the referee very much for the comments and suggestions. They are very helpful for us to revise and improve the paper. The paper has been carefully revised according to the referee’s advice. We have made the following changes on the paper accordingly.

**Reviewer #1, Concern #1:** In the main text, there is some error for the format of the reference.

**Author response:** Thank you for your valuable feedback and for taking the time to review our manuscript. We sincerely appreciate your thorough evaluation, which has provided us with valuable insights to improve the quality of our work.

As suggested by the reviewer, The format errors of references in the main text have been corrected.

The changes have been marked in revision mode, and the revised content is also indicated in yellow in the manuscript, which we have attached for your convenience.

**Reviewer #1, Concern #2:** Some writing error exists in the paper. Please check the full text carefully and revise them.

In Eq. 7, what is ‘$w_{l}^{A}$’? Should it be ‘$w_{l}^{Z}$’.

In page 28, ‘As shown in Fig. 7, this paper sets the parameters of the original GA involved in the comparison to two cases,’ the ‘Fig.7’ may be ‘Fig. 12’.

**Author response:** Thank you very much for reviewing our paper and providing valuable feedback. We sincerely appreciate your thorough evaluation, which has provided us with valuable insights to improve the quality of our work.

In Eq. 7, the ‘$w_{l}^{A}$’ should be ‘$w_{l}^{Z}$’, it is the relative importance of customer requirements based on the customer requirements importance judgment matrix in the Eq. 7.

In page 28, ‘As shown in Fig. 7, this paper sets the parameters of the original GA involved in the comparison to two cases,’ the ‘Fig.7’ should be ‘Fig. 11’, it is the comparison result of IGA and GA.

The changes have been marked in revision mode, and the revised content is also indicated in yellow in the manuscript, which we have attached for your convenience.

**Reviewer #1, Concern #3:** In the case study, the example studied the living room with an area of 30 square meters and located in Shenyang City, Liaoning Province, China. Besides, The author stated that they investigated 148 adult customers to obtain related data used in this paper. Please give a detailed illustration about these 148 customers to testify the rationality of selecting these 148 adult customers, such as the relationship between them.

**Author response:** Thank you very much for reviewing our paper and providing valuable feedback. We sincerely appreciate your thorough evaluation, which has provided us with valuable insights to improve the quality of our work.

In order to testify the rationality of selecting these 148 adult customers, the detailed illustration about them has been given in the section “6.1 Quantification of the relative importance weight of customer requirements”.

The changes have been marked in revision mode, and the revised content is also indicated in yellow in the manuscript, which we have attached for your convenience.

**Reviewer #1, Concern #4:** In table 12, how did the authors determine the value of the parameters illustrated in section 4.2? For example, the ‘g1’ in Eq. 8 is valued as ‘g1=2’ in table 12, and so on.

**Author response:** Thank you very much for reviewing our paper and providing valuable feedback. We sincerely appreciate your thorough evaluation, which has provided us with valuable insights to improve the quality of our work.

According to referee’s comments, we have supplemented the explanation of "how to determine the utility measure function types of each candidate itemset and the parameter node values in it in case analysis" in the back of Table 12.

The changes have been marked in revision mode, and the revised content is also indicated in yellow in the manuscript, which we have attached for your convenience.

**Reviewer #1, Concern #5:** In section 6.5, the author provided the performance analysis by comparison result of IGA and GA, where original GA with two different parameter settings. However, these two parameter settings are different with the one used in IGA. The author is advised to add an experimental comparison with GA that has the same parameter settings used in IGA shown in Table 14 to increase the credibility of the results.

**Author response:** Thank you very much for reviewing our paper and providing valuable feedback. We sincerely appreciate your thorough evaluation, which has provided us with valuable insights to improve the quality of our work.

In section 6.5, the parameter setting of GA-2 is same with the parameter setting of IGA in Table 14. There was a writing mistake in the previous manuscript, which has now been corrected.

The changes have been marked in revision mode, and the revised content is also indicated in yellow in the manuscript, which we have attached for your convenience.

**Response to the Comments of Reviewer #2 on PONE-D-24-37417**

We thank the referee very much for the comments and suggestions. They are very helpful for us to revise and improve the paper. The paper has been carefully revised according to the referee’s advice. We have made the following changes on the paper accordingly.

**Reviewer #2, Concern #1:** Provide a more brief real-world example early on to illustrate the practical significance of this research problem.

**Author response:** Thank you for your valuable feedback and for taking the time to review our manuscript. We sincerely appreciate your thorough evaluation, which has provided us with valuable insights to improve the quality of our work.

According to referee’s comments, we have already provided a more brief real-world example in the first paragraph of section 3 “Problem description” to illustrate the practical significance of this research problem.

The changes have been marked in revision mode, and the revised content is also indicated in green in the manuscript, which we have attached for your convenience.

**Reviewer #2, Concern #2:** The description of the proposed approach in Section 4 could benefit from a high-level overview or flowchart before diving into the detailed steps.

**Author response:** Thank you for reviewing our manuscript and providing valuable feedback. We sincerely appreciate your thorough evaluation, which has provided us with valuable insights to improve the quality of our work.

According to referee’s comments, in Section 4, we have already added a flow chart named “Fig. 3” to describe specific process of service resource configuration optimization before diving into the detailed steps

The changes have been marked in revision mode, and the revised content is also indicated in green in the manuscript, which we have attached for your convenience.

**Reviewer #2, Concern #3:** How might this method be adapted or extended to account for dynamic customer preferences that may change over time?

**Author response:** Thank you for your valuable feedback and for taking the time to review our manuscript. We sincerely appreciate your thorough evaluation, which has provided us with valuable insights to improve the quality of our work.

As suggested by the reviewer, in Section 7 " Conclusion and discussion", we have taken the issue of " dynamic customer preferences that may change over time can be introduced into service resource configuration optimization" as the content of further research in the future.

The changes have been marked in revision mode, and the revised content is also indicated in green in the manuscript, which we have attached for your convenience.

**Reviewer #2, Concern #4:** Elaborate on the broader implications and potential applications of this research in other domains beyond living room customization.

**Author response:** Thank you very much for reviewing our paper and providing valuable feedback. We sincerely appreciate your thorough evaluation, which has provided us with valuable insights to improve the quality of our work.

In response to the reviewers' feedback, in the fifth paragraph of Section 7 " Conclusion and discussion", we added the broader implications and potential applications of this research in other domains beyond living room customization.

The changes have been marked in revision mode, and the revised content is also indicated in green in the manuscript, which we have attached for your convenience.

**Reviewer #2, Concern #5:** In what ways could machine learning or artificial intelligence techniques be integrated into this approach to further enhance its capabilities?.

**Author response:** Thank you very much for reviewing our paper and providing valuable feedback. We sincerely appreciate your thorough evaluation, which has provided us with valuable insights to improve the quality of our work.

As suggested by the referee, this study can introduce the application of machine learning and artificial intelligence technology to improve the accuracy and effectiveness of the research results. For example, in the service requirement analysis, machine learning technology can be used to capture and analyze the online reviews of corresponding products, so as to better obtain and control the diversified requirements of customers; In the part of algorithm solution, machine learning technology can also be introduced for better parameter optimization.

The changes have been marked in revision mode, and the revised content is also indicated in green in the manuscript, which we have attached for your convenience.

**Reviewer #2, Concern #6:** Break up some of the longer paragraphs into shorter ones to improve readability.

**Author response:** Thank you very much for reviewing our paper and providing valuable feedback. We sincerely appreciate your thorough evaluation, which has provided us with valuable insights to improve the quality of our work.

In response to the reviewers' feedback, we have break up some of the longer paragraphs into shorter ones to improve readability.

The changes have been marked in revision mode, and the revised content is also indicated in red in the manuscript, which we have attached for your convenience.

**Response to the Comments of Reviewer #3 on PONE-D-24-37417**

We thank the referee very much for the comments and suggestions. They are very helpful for us to revise and improve the paper. The paper has been carefully revised according to the referee’s advice. We have made the following changes on the paper accordingly.

**Reviewer #3, Concern #1:** The title effectively reflects the content and focus of the research. The abstract is informative, providing a concise summary of the problem, proposed solution, and methodologies used. It mentions key elements such as the KANO model, HoQ, and genetic algorithm, all relevant to the topic. However, the readability of the abstract could be improved for better flow.

**Author response:** Thank you for your valuable feedback and for taking the time to review our manuscript. We sincerely appreciate your thorough evaluation, which has provided us with valuable insights to improve the quality of our work.

In response to the reviewers' feedback, we have added some logical relational words in the abstract to improve the readability of the article.

The changes have been marked in revision mode, and the revised content is also indicated in blue in the manuscript, which we have attached for your convenience.

**Reviewer #3, Concern #2:** The introduction provides a strong foundation by addressing the growing importance of personalized customization in today's market. It clearly explains the shift from a product-oriented to a service-oriented business model. However, some parts are overly verbose, and there is some repetition in discussing the problem and research objectives. The identification of the research gap—previous studies lacking a "oneto-many" relationship mechanism between service modules and candidate itemsets—is particularly strong. The objectives are clear, though the introduction would benefit from a more explicit research question or hypothesis.

**Author response:** Thank you for your valuable feedback and for taking the time to review our manuscript. We sincerely appreciate your thorough evaluation, which has provided us with valuable insights to improve the quality of our work.

According to referee’s comments, we have simplified and deleted some repetitive and lengthy paragraphs or contents in this article. For example, in the Section 1 “Introduction”, we have simplified and split some long paragraphs; And the repeated description of the "one-to-many" relationship mechanism in the last paragraph of Section 2 “Related work” has been deleted.

The changes have been marked in revision mode, and the revised content is also indicated in blue in the manuscript, which we have attached for your convenience.

**Reviewer #3, Concern #3:** The review covers the relevant literature on service requirement analysis, modularization, and service configuration. However, some references appear outdated or tangential to the core contribution. The research gap is well-articulated, though the review could better highlight how the proposed work advances or differs from existing approaches.

**Author response:** Thank you for your valuable feedback and for taking the time to review our manuscript. We sincerely appreciate your thorough evaluation, which has provided us with valuable insights to improve the quality of our work.

According to referee’s comments, we have deleted and updated some references appear outdated or tangential to the core contribution. In Section 4, we have already added a flow chart named “Fig. 3” to describe specific process of service resource configuration optimization before diving into the detailed steps.

The changes have been marked in revision mode, and the revised content is also indicated in blue in the manuscript, which we have attached for your convenience.

**Reviewer #3, Concern #4:** The paper introduces the importance judgment matrix, the KANO model, and the HoQ, which are wellsuited for evaluating customer needs and service modules. However, the explanation of the mathematical model could be more concise and accessible for readers unfamiliar with these techniques. The use of an improved genetic algorithm (IGA) is well-justified, particularly for handling large-scale optimization. Further details on how the IGA improves upon traditional genetic algorithms would add clarity. The case study on living room customization is useful, though breaking it into smaller, more manageable sections would improve reader comprehension. I would recommend including a more straightforward or introductory example before delving into the living room customization example. This would help readers grasp the core concepts before dealing with a more complex scenario.

**Author response:** Thank you for your valuable feedback and for taking the time to review our manuscript. We sincerely appreciate your thorough evaluation, which has provided us with valuable insights to improve the quality of our work.

According to referee’s comments, we have explained some further details of the IGA; In the Section 6 “Case study”, we have added a more straightforward or introductory example before delving into the living room customization example.

The changes have been marked in revision mode, and the revised content is also indicated in blue in the manuscript, which we have attached for your convenience.

**Reviewer #3, Concern #5:** The quantitative analysis appears thorough, though some aspects of the results section seem rushed. Greater emphasis on the real-world implications of the results, particularly customer satisfaction, would strengthen the analysis. The inclusion of various utility functions is commendable, but clearer justification for the selection of specific functions for different service modules would aid in understanding.

**Author response:** Thank you for your valuable feedback and for taking the time to review our manuscript. We sincerely appreciate your thorough evaluation, which has provided us with valuable insights to improve the quality of our work.

As the referee said, in the sixth and seventh paragraphs of the Section 1 “Introduction”, we have explained clearer justification for the selection of specific functions for different service modules when meeting different customer requirements.

The changes have been marked in revision mode, and the revised content is also indicated in blue in the manuscript, which we have attached for your convenience.

**Reviewer #3, Concern #6:** The discussion provides insightful reflections on the model's effectiveness. However, it could benefit from more direct comparisons to alternative approaches from the literature. The paper does not explicitly address the limitations of the approach. A discussion of potential weaknesses or areas for improvement would enhance the rigor of the analysis—such as scalability to other industries including a brief discussion of computational costs or the complexity of scaling up the model. It could also benefit from a discussion of specific user-centric metrics or KPIs (Key Performance Indicators): how are the improvements in customer satisfaction quantitatively measured and compared to other methods? You may also consider adding a discussion on how the model could adapt to emerging trends in customization, such as the use of AI in real-time customization, or integration with cloud-based services for better data management. Future-proofing the methodology could increase its long-term relevance.

**Author response:** Thank you for your valuable feedback and for taking the time to review our manuscript. We sincerely appreciate your thorough evaluation, which has provided us with valuable insights to improve the quality of our work.

According to referee’s comments, in the fifth paragraph of Section 7 " Conclusion and discussion", we have added the broader implications and potential applications of this research in other industries beyond living room customization.

This study can introduce the application of machine learning and artificial intelligence technology to improve the accuracy and effectiveness of the research results. For example, in the service requirement analysis, machine learning technology can be used to capture and analyze the online reviews of corresponding products, so as to better obtain and control the diversified requirements of customers; In the part of algorithm solution, machine learning technology can also be introduced for better parameter optimization. These are also the key points that can be studied in the next step of this paper, which have been pointed out in the seventh paragraph of Section 7 " Conclusion and discussion".

The changes have been marked in revision mode, and the revised content is also indicated in blue in the manuscript, which we have attached for your convenience.

**Reviewer #3, Concern #7:** The conclusion effectively summarizes the key contributions of the paper but could be more succinct. Suggestions for future research are implied but not fully developed. Expanding on specific areas for further exploration, such as integrating real-time data or applying the model to different customization domains, would strengthen the conclusion.

**Author response:** Thank you for your valuable feedback and for taking the time to review our manuscript. We sincerely appreciate your thorough evaluation, which has provided us with valuable insights to improve the quality of our work.

According to referee’s comments, we have simplified the conclusion; in the fifth paragraph of Section 7 " Conclusion and discussion", we have added the broader implications and potential applications of this research in other industries beyond living room customization.

The changes have been marked in revision mode, and the revised content is also indicated in blue in the manuscript, which we have attached for your convenience.
