# Supplementary material for: Personalized customization: Service resource configuration optimization driven by customer requirements accurately
Source: PLoS One. 2025 Apr 14;20(4):e0320312. doi: 10.1371/journal.pone.0320312 (PMC11996078; doi:10.1371/journal.pone.0320312)
Supplement: Attachment — Submitted filename: comments.docx [file pone.0320312.s002.docx]

The paper develops an approach of evaluating and optimizating the service resource configuration in the context of personalized customization, where an evaluation method for the relative importance of customer requirements and improved genetic algorithm for the service resource configuration were proposed, and the viability and efficacy of the approach were demonstrated by an example of living room customization in a customization company. The research has important significance in both theory and practice for the for the importance analysis of customer requirements, but it still has some issues that the authors need to address to improve the quality of the paper.

(1) In the main text, there is some error for the format of the reference.

(2) Some writing error exists in the paper. Please check the full text carefully and revise them.

In Eq. 7, what is ‘wA-l’? Should it be ‘wZ-l’.

In page 28, ‘As shown in Fig. 7, this paper sets the parameters of the original GA involved in the comparison to two cases,’ the ‘Fig.7’ may be ‘Fig. 11’.

(3) In the case study, the example studied the living room with an area of 30 square meters and located in Shenyang City, Liaoning Province, China. Besides, The author stated that they investigated 148 adult customers to obtain related data used in this paper. Please give a detailed illustration about these 148 customers to testify the rationality of selecting these 148 adult customers, such as the relationship between them.

(4) In table 12, how did the authors determine the value of the parameters illustrated in section 4.2? For example, the ‘g1’ in Eq. 8 is valued as ‘g1=2’ in table 12, and so on.

(5) In section 6.5, the author provided the performance analysis by comparison result of IGA and GA, where original GA with two different parameter settings. However, these two parameter settings are different with the one used in IGA. The author is advised to add an experimental comparison with GA that has the same parameter settings used in IGA shown in Table 14 to increase the credibility of the results.
